# Machine learning-based prediction of tensile strength of glass fiber-reinforced polymer rebar under environmental conditions

**DOI:** 10.1177/13694332251363357

**Published:** 2025-07-29

**Authors:** Yuqing Cheng, Xiangdong Geng, Chao Wu

**Affiliations:** 1 School of Transportation Science and Engineering, 12633 Beihang University, Beijing, China; 2 Department of Civil and Environmental Engineering, 4615 Imperial College London, UK

**Keywords:** GFRP rebar, machine learning, durability, tensile strength, statistical analysis

## Abstract

GFRP rebar is a desirable alternative to steel rebar especially in harsh environments with advantages of light weight, high strength, stable chemical properties, and corrosion resistance. However, there is a lack of understanding on the durability of GFRP rebar which significantly limits its engineering applications. Unfortunately, it is almost impossible to develop any simple theoretical model to predict the residual strength of GFRP rebar after environmental exposure. To effectively address this research gap, this paper investigates machine learning models to predict the residual tensile strength of GFRP rebar due to environmental degradation. Firstly, a database was built from the literature containing a total of 350 tensile testing results of GFRP rebar after experimental exposure. Six key influencing parameters were considered, including fiber content, bar diameter, resin type, exposure temperature, pH, and aging time. Secondly, machine learning models were trained and tested using the database, and the selected models included Decision Tree, Random Forest, Support Vector Machine (SVM), Multilayer Perceptron (MLP), and Long and Short-Term Memory (LSTM) models. The LSTM model demonstrated the best performance in strength prediction, achieving an R^2^ of 0.96 on the training set and 0.91 on the testing set. Thirdly, the influencing parameters were ranked using SHAP and Random Forest in terms of their impact on the residual tensile strength of GFRP rebar, and it was found that temperature has the most significant effect followed by fiber content, exposure time, pH and bar diameter, while resin type showed the least importance. Notably, SHAP analysis also showed that the coupling of different parameters also had impact on the residual strength, and the combined effect of fiber content with other parameters was the most prominent. This paper demonstrates the feasibility of machine learning models in the durability study of GFRP composite materials.

## Introduction

Fiber reinforced composites (FRP) are widely used in aerospace, automotive and civil engineering due to their desirable properties like light weight, high strength and corrosion resistance (Rubino et al., 2020; Subbaya and Nithin, 2017; Yang et al., 2019). FRP rebars are compelling alternatives to traditional steel rebars due to their corrosion resistance in harsh environments which is desirable for improving the service life of infrastructure (Rubino et al., 2020). Notably, Glass Fiber Reinforced Polymer (GFRP) rebars have emerged as the predominant choice due to their low production cost, well-established manufacturing technology, and chemical stability (Nkurunziza et al., 2005; D'Antino et al., 2018).

GFRP rebars may degrade in tensile strength under harsh service environments. For example, it was found (Al-Salloum et al., 2013; Wu et al., 2022) that the GFRP tensile strength may decrease under temperature and alkalinity exposures. It was reported (Amaro et al., 2013; Rocha et al., 2017) that the resin matrix of GFRP rebars is more vulnerable to environmental effects than the glass fibers. Resin undergoes plasticization due to moisture absorption leading to swelling, molecular change, and microcracks, which negatively affects the mechanical properties of the rebars.

The strength reduction of GFRP rebars is complex and affected by many factors. In real applications, the bars are exposed to different environmental conditions which may be coupled. This coupling effect cannot be captured by traditional accelerated durability testing in the lab. It is extremely challenging to predict strength reduction of GFRP rebars using traditional lab testing and theoretical modelling. On the other hand, machine learning emerges as a powerful tool for regression and classification problems in civil engineering (Baghaei and Hadigheh, 2021; Feng et al., 2020; Yin and Liew, 2021). For example, Baghaei and Hadigheh (2021) used various machine learning models such as decision tree, support vector machine (SVM), and artificial neural network (ANN) to predict the bond strength between FRP and concrete in wet environment, and they found that ANN has the best performance. Yin and Liew (2021) evaluated the interfacial shear strength and maximum pull-out force of FRP composites using five machine learning models. They found that the effect of each factor on these interfacial properties was nonlinear, and the interfacial shear strength was affected by the fiber diameter the most (Yin and Liew, 2021). Feng et al. (2020) used an integrated algorithm to predict the compressive strength of concrete. It was found that the AdaBoost integrated algorithm was the most efficient and accurate, and it was also able to capture the changes in compressive strength due to different curing time.

Recurrent class neural network (RNN) has been developed for time series problems (Medsker and Jain, 1999; Sherstinsky, 2020). Compared with traditional neural networks, RNN has a simple memory function, which can store the temporal information in the data and transfer it inside the network. However, traditional RNN model suffers from gradient vanishing and explosion, which severely limits its effectiveness for long sequence problems. To overcome this issue, Hochreiter and Schmidhuber (1997) proposed the Long Short-Term Memory Neural Networks (LSTM), which incorporates a gating mechanism to effectively capture and regulate information over long sequences, thereby mitigating gradient-related issues.

LSTM model has been successfully used to predict the strength of materials. Zhang et al. (2019) investigated the fused deposition molding (FDM) printing process, and used LSTM model to predict the tensile strength of the manufactured parts considering the other material properties and printing parameters. The results confirmed that LSTM was effective in modelling the sequential layer-by-layer FDM process, and found that the extrusion temperature, printing speed and layer height had a greater impact on the tensile strength prediction. Chen et al. (2022) predicted the compressive strength of high-strength concrete with machine learning models and investigated the effect of parameters including cement ratio, aggregate, and water reducer on the compressive strength. LSTM was found to have higher accuracy and reliability than other machine learning models.

This paper aims to predict the residual tensile strength of GFRP rebars after exposure to complex environmental conditions using machine learning models. A database was established using experimental results in the literature. Machine learning models like Decision Tree, Random Forest, Support Vector Machine (SVM), Multilayer Perceptron (MLP), and Long and Short-Term Memory (LSTM) models were trained and tested using the database. The effectiveness and accuracy of these machine learning models were compared. The key parameters affecting the tensile strength of GFRP rebars were evaluated and ranked using SHAP and Random Forest methods. The correlation of influencing parameters and its impact on tensile strength of GFRP rebar was investigated using SHAP based on LSTM model. It was found that LSTM showed the best performance to predict the residual tensile strength of GFRP rebar. According to the SHAP values based on LSTM model, the most important parameters affecting the tensile strength were exposure temperature and fiber content, followed by exposure time, pH and bar diameter, while resin type showed the least importance. The correlation between influencing parameters also had impact on the tensile strength, and it was found that the combined effect of fiber content with other parameters was the most prominent. This paper demonstrates the important role and significant potential of machine learning as a powerful tool for durability study of GFRP rebars.

## Database

### Database description

The tensile strength of GFRP rebar is defined as the maximum tensile stress the material can resist per unit cross-sectional area before failure, serving as a fundamental indicator of the load-bearing capacity in structural application. The parameters affecting the tensile strength of GFRP rebar can be divided into two categories (Liu et al., 2020), material properties of GFRP rebar and environmental conditions. Existing laboratory studies primarily focus on humid environment and alkaline condition. This study collects experimental data from accelerated testing of GFRP rebars. A total of 350 valid experimental data points (Al-Salloum et al., 2013; Benmokrane et al., 2017; Chen et al., 2006, 2007; Davalos et al., 2012; El-Hassan et al., 2017, 2018; He et al., 2013, 2017; Kim et al., 2008; Micelli and Nanni, 2004; Prian et al., 1997; Robert and Benmokrane, 2013; Robert et al., 2009; Sawpan et al., 2014; Won et al., 2008) from published papers are selected, including six influencing parameters as presented in Table 1. The collected experimental results are presented in the Appendix Table of this paper. The influencing parameters include the properties of GFRP rebar, that is mass fraction of fiber content (FC), bar diameter (d), resin type, and environmental factors such as temperature (T), environment pH (pH), exposure time (t). Four types of resin are considered including epoxy, vinyl ester, polyester, and thermoplastics. As initial tensile strength of GFRP rebar vary across different studies, making direct comparison of environmental effects challenging. To overcome this issue, this study uses residual tensile strength (RS) which is defined as the strength drop after environmental exposure divided by the initial bar strength, as shown in the following formula.

(1) $$RS=\frac{T_{i}-T_{w}}{T_{i}}$$

Where, $T_{i}$ and $T_{w}$ denote the tensile strength before and after exposure, respectively. Tensile tests of GFRP rebar before and after environmental exposure are normally conducted in accordance with ASTM D3916-94 standard (2017).

**Table 1.** Literature source of database and the selected key influencing parameters.

	Reference	Resin type	Diameter (mm)	Fiber content (wt.%)	Environment alkalinity	Temperature (°C)	Exposure time (h)
1	Chen et al. (2007)	Vinyl ester	9.53	-	Tap water/Alkaline solution (pH = 13.6/13/12.7)	40°C/60°C	1680
2	Robert and Benmokrane (2013)	Vinyl ester	12.7	77.9	Seawater and cement (pH = 12.15)	23°C/40°C/50°C/70°C	1440-8760
3	He et al. (2017)	Vinyl ester	10	72	Tap water/Alkaline solution (pH = 13.5)	23°C/60°C	4320-12960
4	Micelli and Nanni (2004)	Thermoplastic, Polyester	12/6.35	-	Alkaline solution (pH = 12.6-13.0)	60°C	504-2336
5	Robert et al. (2009)	Vinyl ester	12.7	81.5	Cement (pH = 12.15)	23°C/40°C/50°C	1440-5760
6	El-Hassan et al. (2018)	Epoxy	7.2/8/9	78,75	Seawater and cement	20°C/40°C/60°C	1800-10800
7	Chen et al. (2006)	Vinyl ester	9.53	70	Alkaline solution (pH = 12.7,13.6)	20°C/40°C/60°C	1440-5760
8	Benmokrane et al. (2017)	Vinyl-ester	9.5/12.7/15.9/19.1/25.4	80.9/81.8/82.6/82.7/83	Alkaline solution (pH = 12.6)	60°C	2160
9	Davalos et al. (2012)	Vinyl ester	9.53	70	Cement	20°C/40°C/50°C/60°C	720-6480
10	El-Hassan et al. (2017)	Epoxy	7.2/8/9	78.3/75.5	Seawater and cement	20°C/40°C/60°C	3600-10800
11	Kim et al. (2008)	Vinyl ester	12.7	73.3/69.1	Tap water/Seawater/ Alkaline solution (pH = 13)	25°C/40°C/80°C	720-3168
12	He et al. (2013)	Vinyl ester	10.2	51	Cement	23°C/60°C	1440-25920
13	Won et al. (2008)	Vinyl ester	12.7	-	Alkaline solution (pH = 12.6)	20°C/40°C/60°C/80°C	720-7200
14	Prian et al. (1997)	Polyester, Vinyl ester	6.35	50/60	Water/Alkaline solution (pH = 12)	20°C/50°C/80°C	336-672
15	Sawpan et al. (2014)	Vinyl ester	14	-	Alkaline solution (pH = 13)	60°C	744-17520
16	Al-Salloum et al. (2013)	Vinyl ester	12	83	Water/seawater/Alkaline solution (pH = 12.5-13)	Room temperature/50°C	4380-13140

For the six key parameters in Table 1, the published papers may not report all of them. Research find missing data can lead to biased analysis, inaccurate predictions, and overall performance degradation in machine learning models. When the proportion of missing data is high, it can distort relationships between variables, leading to misinformed conclusions (Ayilara et al., 2019; Emmanuel et al., 2021). Furthermore, missing data mechanisms can be categorized into Missing Completely at Random (MCAR), Missing at Random (MAR), and Not Missing at Random (NMAR). The collected GFRP rebar tensile strength data follows the MAR mechanism, meaning that the probability of missing data depends on the observable variables but is independent of the missing data itself. In the study of GFRP reinforcement, factors such as resin type, fiber content, and environmental temperature are objective and do not change due to the recording process. Therefore, for MAR data, applying appropriate imputation methods can effectively improve data quality, thereby enhancing the predictive performance of machine learning models (Ayilara et al., 2019; Emmanuel et al., 2021). Plaia and Bondi (2006) applied a simple imputation method to reconstruct monitored meteorological data and studied the impact of imputation methods on data quality. The results showed that a simple imputation method incorporating multiple types of information effectively restored the missing data.

Therefore, a simple interpolation method is used to complement the missing values in literature by considering the attributes of the feature types. For the fiber content in weight percentage, the value is estimated based on the resin type and bar diameter. For example, for GFRP rebar with a diameter of 12.7 mm and with a resin of vinyl ester, three fiber content values are reported that is73.3%, 81.5%, and 77.9%. Therefore, if fiber content is not reported, an average value that is 77.566% is used to fill in the database. For the environmental alkalinity, if GFRP rebar is embedded in concrete, a pH value of 13.7 is chosen. If the rebar is immersed in seawater, a pH value of eight is adopted considering the polyionic environment. If the GFRP rebar is immersed in deionized water or tap water, the pH value is set to 7. For temperature, 23°C is used for room temperature condition. The statistical results of the key parameters in the database are shown in Table 2. Except for the exposure time, the standard deviation of each parameter is small. In general, the rebars are subjected to alkaline condition with an average exposure temperature of 43.8°C. It should noted that the database in Table 2 is used to train the machine learning models in this paper. In other words, the performance of machine learning models is assessed with the parameters satisfying the database in Table 2. If the parameters are beyond the ranges in Table 2, the performance of machine learning models cannot be guaranteed.

**Table 2.** Statistical results of key parameters in the database.

	Max	Min	Mean	Standard deviation	Variation coefficient
Fiber content (wt.%)	83	50	73.123	7.001	0.096
Diameter (mm)	25.4	6.35	10.510	2.360	0.225
pH	13.7	2.88	11.608	2.777	0.239
Temperature (°C)	80	20	43.8	18.653	0.426
Exposure time (h)	25920	336	4354.217	3628.105	0.833
Residue strength	1.11	0.23	0.782	0.193	0.247

### Data regularization

Due to the varying units of different influencing parameters, their absolute values have significant disparity. For instance, the maximum exposure time is 25,920 hours, whereas the pH has a minimum value of just 2.33. Such a large-scale difference can affect the accuracy of the machine learning model and slow down its convergence. Therefore, it is essential to regularize the data.

Common regularization methods include Min-Max Scaling and Z-score Standardization. The Min-Max Scaling method scales data within a specified range, typically [0,1], based on the difference between the maximum ( $x_{\max}$ ) and minimum ( $x_{\min}$ ) values for a particular parameter. Z-score Standardization measures the distance of each data deviation from the data mean ( $\bar{x}$ ) with the standard deviation ( σ ), and transforms the data into a standard distribution with a mean of 0 and a variance of 1, thereby reducing the impact of outliers on the model and enhancing its robustness. The formulas for both methods are presented below.

(2) $$x_{\min-\max}=\frac{x-x_{\min}}{x_{\max}-x_{\min}}$$

(3) $$x_{z-score}=\frac{x-\bar{x}}{\sigma }$$

Given the scattered distribution of the collected data and in order to minimize experimental errors and the impact of outliers on the model, the Z-score Standardization method is employed in this paper. This is implemented using the Standard Scaler function from the scikit-learn library in Python, with the results shown in Table 3. For categorical data such as resin type, one-hot encoding is used to convert it into a matrix for model input. In the database, there are four types of resin: epoxy, vinyl ester, polyester, and thermoplastics. Using one-hot coding for the type of resin, it will be transformed into a matrix of 350 × 4, where 350 is the total number of data points studied and four is the number of resin types, with each column representing a type of resin. For example, for a GFRP rebar using vinyl ester, the coding will be transformed into an array of [0,1,0,0], where the second column represents the type of matrix used as vinyl ester, and the first, third, and fourth columns respectively represent polyester, epoxy, and thermoplastic resins. In the following text, E-P, E-V, E-E, and E-T correspond to four types of resin: polyester, vinyl ester, epoxy, and thermoplastic resin.

**Table 3.** Statistical results after regularization.

	Max	Min	Mean	SD
Fiber content (wt.%)	1.41	−3.3	0.000184571	1.00
Diameter (mm)	6.31	−1.76	0.000794286	1.00
pH	0.753	−3.14	−0.000525714	1.00
Temperature (°C)	1.94	−1.28	−0.001651429	1.00
Exposure time (h)	5.94	−1.11	0.000129143	1.00

## Machine learning modelling

Machine learning models generally handle problems in two main categories: classification and regression. Predicting the residual tensile strength of GFRP rebars falls into the regression category. Decision Tree and Random Forest, as representative tree-based models, provide strong interpretability and are effective in handling structured tabular data. Support Vector Machine (SVM), a kernel-based algorithm, performs well in small-sample and high-dimensional scenarios with nonlinear relationships. The Multilayer Perceptron (MLP), a type of feedforward neural network, offers robust nonlinear modeling capabilities with relatively simple architecture. Long Short-Term Memory (LSTM), as an advanced recurrent neural network, is particularly suited for modeling temporal dependencies in sequential data.

Based on these complementary strengths, Decision Tree, Random Forest, SVM, MLP, and LSTM were selected as the machine learning models for predicting the residual tensile strength of GFRP rebars.

### Decision tree, Random Forest, and support vector machine (SVM) models

This paper employs Decision Tree, Random Forest, and Support Vector Machine (SVM) models because they are the most used representative machine learning models. Decision Tree (Myles et al., 2004) is a tree-structured machine learning model that builds a tree through a series of decision nodes, each representing a feature (influencing parameters in this paper) to divide the dataset into different subsets. At each node, the model makes a decision based on the feature’s condition to choose the next node until reaching a leaf node, which contains the final category label or regression value. The performance of a decision tree is determined by hyperparameters related to the tree. A common decision tree model is the CART (Classification and Regression Trees) algorithm, which constructs a complex decision tree first and then prunes it according to the data outcome to achieve optimal results.

**Figure 1.**
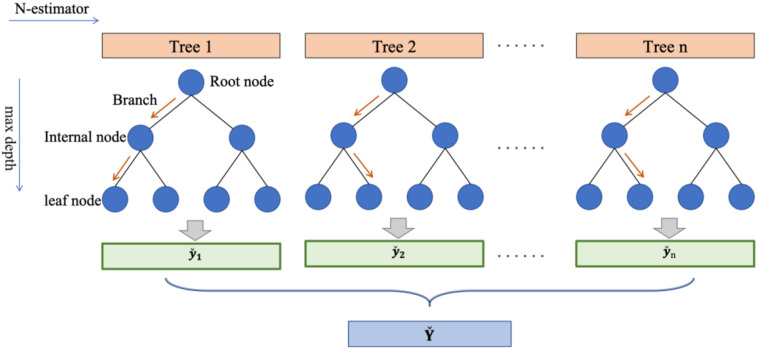
Structure of the Random Forest model.

Random Forest (Breiman, 2001) is an ensemble model composed of many decision trees, as shown in Figure 1. For any given tree n, it generates independent, identically distributed random vector $\Theta_{n}$ and uses the training set along with $\Theta_{n}$ to build the tree model n. These trees are then combined to form a Random Forest. There are different methods to construct random matrices, for example bagging method uses repetitive sampling to establish *Θ*, random splitting builds *Θ* by randomly selecting features and their combinations. The common goal of these methods is to introduce randomness, create diversified trees, and combine these trees to enhance the global performance of the Random Forest model.

Random Forest follows the CART algorithm of Decision Tree for feature selection and division but differs in two main aspects. Firstly, in feature selection, while Decision Tree chooses the best feature from all available features at each split, Random Forest selects the best feature from a subset of randomly chosen features. Secondly, in terms of training data, Random Forest uses the bagging method, where multiple subsets of the original training data are obtained through sampling with replacement, and multiple decision trees are independently trained. The final prediction is the average of each tree’s results, unlike Decision Trees which often uses the entire dataset. Compared to Decision Tree model, Random Forest reduces the risk of overfitting and enhances generalizability.

Moreover, Random Forest measures the importance of each feature using the entropy reduction method. Each tree in the Random Forest uses features to split data, calculating the entropy used by the feature at each split point. The more the entropy decreases at a node, the more important that feature is considered. Therefore, it can rank the input features in terms of their importance for the output, which is significant for guiding experimental design in this study.

**Figure 2.**
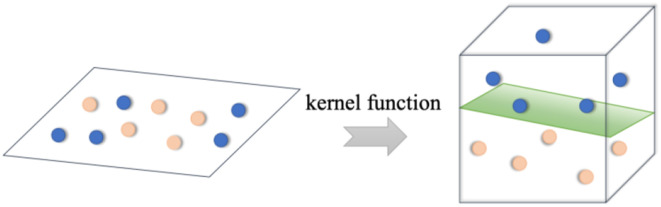
Structure of the SVM model.

Support Vector Machine (SVM) (Noble, 2006) is a powerful supervised learning model that aims to find the decision boundary, or ‘hyperplane’, that maximally separates different categories. SVM introduces kernel functions that map the original data to a higher-dimensional space, transforming nonlinear problems in low dimensions into linear problems in high dimensions, making originally inseparable features in the raw data separable in higher dimensions, as shown in Figure 2.

Common kernel functions include linear kernel (linear), polynomial kernel (polynomial), radial basis function kernel also known as Gaussian kernel (RBF) and sigmoid kernel. Linear kernel is the most basic type of kernel, which is mostly used in linearly separable data with low computational complexity and fast computational speed. x,y is a set of matrix data, and the linear kernel is the dot product between the two. However, it is not ideal for nonlinear data processing.

(4) K(x,y)=x·y

The polynomial kernel adds parameters to the linear kernel to fit more complex data structures, as equation (5).

(5) $$K\left(x,y\right)={\left(\gamma \cdot x\cdot y+r\right)}^{d}$$

Here, γ is the scale parameter, r is the coefficient, and d is the degree of the polynomial. But due to the large number of parameters, it is easy to cause overfitting of the results, and the computational complexity is higher than that of the linear kernel.

The RBF kernel is mostly used for nonlinear data. ‖x−y‖ represents the squared Euclidean distance between x and y . When there is no a priori knowledge of the data, the RBF kernel helps in proper separation compared to other kernels.

(6) $$K\left(x,y\right)=\exp\left({-\gamma \cdot \left\|x-y\right\|}^{2}\right)$$

The Sigmoid kernel is similar to an activation function and is able to map the input data between −1 and 1. However, it does not satisfy the requirement of positive definiteness of the kernel function, which requires that all of the kernel eigenvalues are non-negative, and this can lead to failure in finding the global optimal solution, thus affecting the performance and stability of the model.

(7) K(x,y)=tanh(γ·x·y+r)

SVM model achieves good generalization with suitable kernel functions and proper penalties.

### Deep learning models

Building upon machine learning and with enhanced computing capabilities, deep learning models have evolved to address more complex problems. They have deepened in structure and complexity, forming “end-to-end” fully automated models that do not require manual intervention and can effectively extract features beneficial for solving problems from existing data.

**Figure 3.**
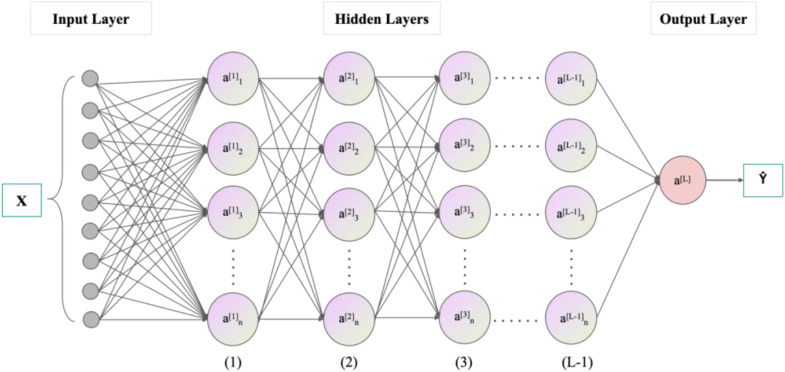
Structure of the MLP model.

Multilayer Perceptron (MLP) model (Pal and Mitra, 1992), developed from Artificial Neural Networks (ANN), is a feedforward neural network. The model consists of an input layer, hidden layers, and an output layer, with each layer containing multiple neurons interconnected. In this study, MLP model for GFRP rebar’s tensile strength prediction includes nine neurons in the input layer and one neuron in the output layer, as shown in Figure 3. Nine neurons in the input layer include the five influencing parameters in Table 3 plus four resin type that is epoxy, vinyl ester, polyester, thermoplastics. The process of information passing from the input layer to the hidden and output layers is called forward propagation, while the process of calculating parameter gradients through the loss function and transmitting them back from the output to the hidden layers is known as backpropagation. The MLP model adjusts itself through iterations of forward and backpropagation to achieve optimal performance.

**Figure 4.**
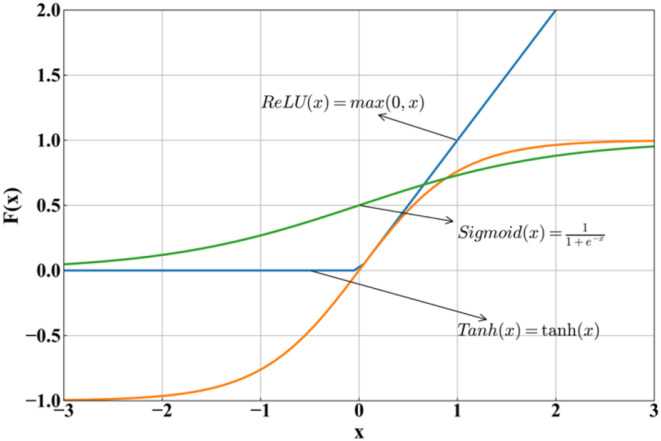
Activation functions.

During the model’s forward propagation, non-linear activation functions are introduced to enhance the model’s ability to learn complex non-linear relationships. Common activation functions include ReLU, Sigmoid, and Tanh. Their formulas and curves are shown in Figure 4. ReLU function could not consider negative parameters values, and Sigmoid function only output positive values. Both of them are not suitable for the database in this paper, which has both negative and positive values of all parameters. Therefore, the Tanh activation function is selected.

Long Short-Term Memory (LSTM) model (Hochreiter and Schmidhuber, 1997) is a type of neural network capable of remembering information for both short and long durations. It has achieved significant success in problems like time series prediction. LSTM is a variant of the Recurrent Neural Network (RNN). Unlike traditional neural networks, RNN introduces memory functions in their hidden layers, allowing them to retain information from previous time steps. However, RNN can suffer from vanishing or exploding gradients when processing long input sequences due to the recurrent multiplication of weight matrices. LSTM addresses these issues by introducing Gated Recurrent Unit (GRU) and memory cells on top of RNN, thereby controlling the retention and transmission of information (Sherstinsky, 2020).

**Figure 5.**
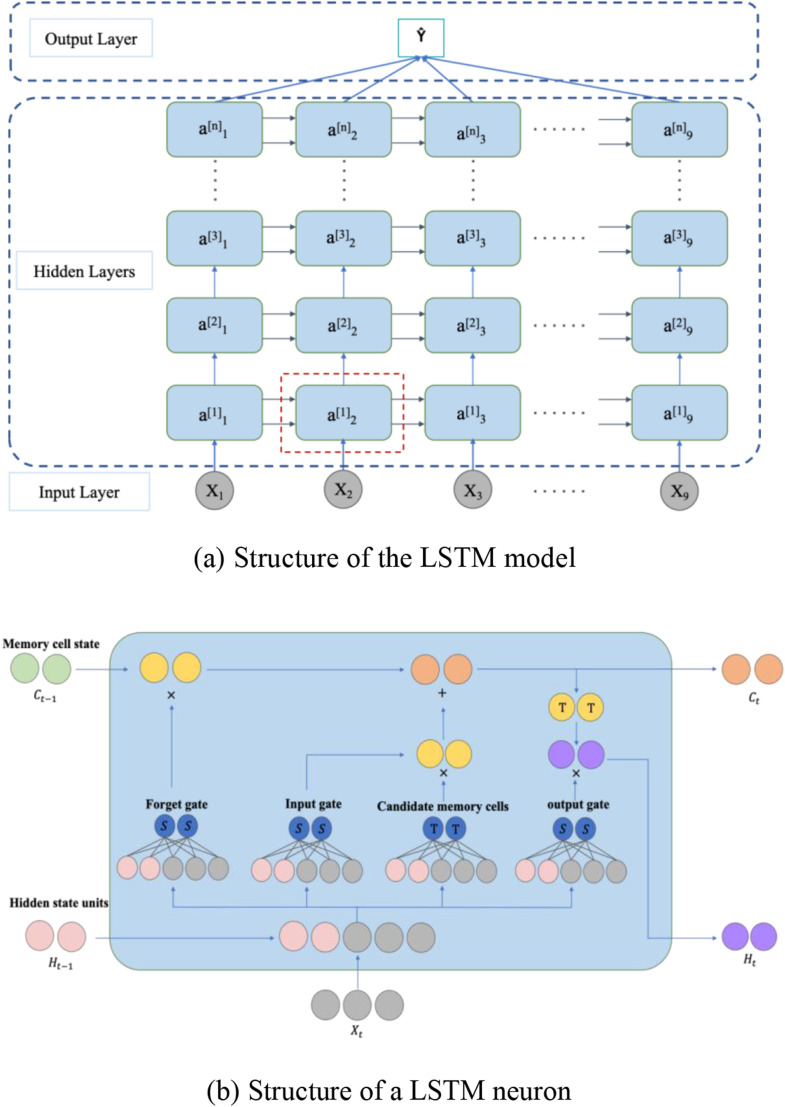
LSTM model. (a) Structure of the LSTM model, (b) Structure of a LSTM neuron.

LSTM has a standard chain-like structure as shown in Figure 5(a). Each repeating unit (or neuron) in Figure 5(b) consists of four network layers, including three gates (input, forget, and output gates) and a memory cell. The input to the gates at each time step includes the current input and the hidden state computed from the previous time step with the output determined by a fully connected layer using a Sigmoid activation function, which ensures gate values ranging between [0,1]. The computational process is as follows:

(1) Gate calculations: the input $x_{t}$ of the current time step is combined with the hidden state $h_{t-1}$ computed in the previous time step through a forgetting gate with Sigmoid as the activation function to get the output between [0,1], which represents the proportion of information to be retained, where 0 means no information is retained and one means all information is retained. The same principle applies to two inputs together through the input gate and output gate to calculate the information to be updated and retained.

(8) $$Forget\;Gate:F_{t}=\sigma \left(W_{f}\left[h_{t-1},x_{t}\right]+b_{f}\right)$$

(9) $$Input\;Gate:I_{t}=\sigma \left(W_{i}\left[h_{t-1},x_{t}\right]+b_{i}\right)$$

(10) $$Output\;Gate:O_{t}=\sigma \left(W_{o}\left[h_{t-1},x_{t}\right]+b_{o}\right)$$

Here, σ represents the Sigmoid activation function, and $\left[h_{t-1},x_{t}\right]$ is the combined input from the previous time step’s hidden state and the current input, with ‘b’ representing the bias.

(2) Calculating the candidate memory cell: using the Tanh activation function, the information that needs to be updated into the memory cells is calculated.

(11) $${\tilde{C}}_{t}=\tanh\left(W_{c}\left[h_{t-1},x_{t}\right]+b_{c}\right)$$

(3) Updating the memory cell: $F_{t}$ calculated by the forgetting gate determines the information passed to the current time step, and the input gate $I_{t}$ controls the information passed to the current time step in the candidate memory cell ${\tilde{C}}_{t}$ . The two are combined as the memory cell for the current time step. And output to the next moment.

(12) $$C_{t}=F_{t}\times C_{t-1}+I_{t}\times {\tilde{C}}_{t}$$

(4) Calculating the hidden state: after obtaining the memory cell $C_{t}$ , the range of the hidden state is guaranteed to be between [-1,1] by the Tanh function, and the result is multiplied with the output gate result to be the final output hidden state $h_{t}$ for that time step. If the output gate result $O_{t}$ is 0, it means that the calculated result is only retained in the memory cell without outputting the hidden state. If it is 1, it completes the data transfer from the memory cell to the hidden state and outputs the hidden state at the same time.

(13) $$h_{t}=O_{t}\times \tanh\left(C_{t}\right)$$

## Modelling results

### Selection of hyperparameters

The paper uses Decision Tree, Random Forest and SVM that comes with the sklearn module in Python. Pytorch is used for coding the MLP and LSTM models. The key hyperparameters in Decision Tree are maximum tree depth, minimum number of samples required to split internal nodes, minimum number of data required per leaf. The hyperparameters are coordinated to achieve the best prediction. For SVM, the important hyperparameters are kernel function type, kernel parameters, and regularization parameters.

To obtain the optimal parameter values, grid search with cross-validation method is used in these machine learning models. Grid search is an exhaustive search method that finds the optimal hyperparameters by traversing the possible combinations of hyperparameters. Cross-validation (CV) is a method for evaluating the performance of a model by dividing the dataset into multiple, non-overlapping training and testing sets for determining the performance of the hyperparameters. The combination of hyperparameters is determined by grid search, and then the performance of the set of parameters on the model is determined by cross-validation, so that the combination of hyperparameters with the best prediction is selected.

The above procedure to determine hyperparameters cannot be used for deep learning models due to the complex model structure, massive neuron data, and high computational cost. It can only be determined by manually adjusting the hyperparameters. The batch size, learning rate, activation function, number of hidden layers, neuron size, and dropout rate are all common hyperparameters for MLP and LSTM models. In addition to the above hyperparameters, the time step hyperparameter in LSTM plays an important role in the model’s processing and understanding of time series data. The time step hyperparameter is integral to the model’s ability to discern dependencies within the dataset, embodying the memory function of the architecture. A protracted time step suggests an enhanced capacity for the model to retain information, thereby enabling it to apprehend more extensive long-term data dependencies. Furthermore, the time step governs the frequency with which data points are incorporated into each input sequence, thereby directly influencing the quantum of data engaged during each iteration of training. This has a consequential impact on both the model’s performance and its training efficiency. By propagating forward and backward at each time step, the model can effectively learn the information in the middle of the sequence and thus perfectly predict the data trend.

In order to accurately predict the residual tensile strength of GFRP rebar, 80% of the database was used for training and the remaining 20% was used for testing the model performance. In addition, 10-fold cross-validation and grid search is used to determine the optimal parameters of the Decision Tree and SVM models. For MLP and LSTM, the optimal parameter values were manually adjusted and determined. The hyperparameter values for each model are presented in Tables 4–8. Please note that the values of hyperparameters in Tables 4–8 are adopted from Python.

**Table 4.** Hyperparameters for decision tree.

Hyperparameter name	Values
Criterion	Squared error function
Max depth	10
Min samples leaf	2
Min samples split	2
Splitter	best

**Table 5.** Hyperparameters for random forest.

Hyperparameter name	Values
Bootstrap	True
Max depth	10
Min samples leaf	2
Min samples split	2
N-estimator	10

**Table 6.** Hyperparameters for SVM.

Hyperparameter name	Values
C	1
Gamma	1
Kernel	rbf

**Table 7.** Hyperparameters for MLP.

Hyperparameter name	Values
Batch size	8
Epoch	150
Learning rate	1e-3
Hidden layer number	3
Neuron number	512
Dropout	0.5
Activation function	Tanh
Loss function	MSE

**Table 8.** Hyperparameters for LSTM.

Hyperparameter name	Values
Batch size	8
Learning rate	0.1
Epoch	200
Loss function	MSE
Layer	6
Dropout	0.7
Activation function	Relu
Neuron number	512

### Prediction performance of different models

**Figure 6.**
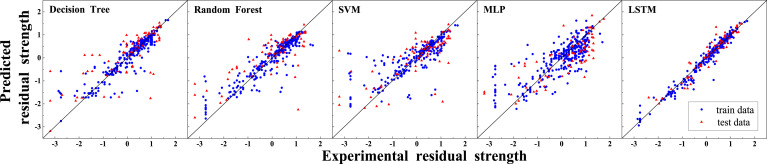
Prediction results of different models.

Figure 6 shows the predicted results of each model. In general, most models are more accurate for positive values and less accurate for negative values. In order to further evaluate the performance of each model, the coefficient of determination (R^2^), the mean square error (MSE), the mean absolute error (MAE), and the root mean square error (RMSE) were chosen as accuracy indicators. The closer R^2^ is to 1, and the smaller MSE, MAE, RMSE is, the more accurate the model is. The accuracy indicators can be calculated using the following equations.

(14) $$R^{2}=1-\frac{\overset{n}{\underset{i=1}{\sum }}{\left(y_{i}-y_{i}^{\prime }\right)}^{2}}{\overset{n}{\underset{i=1}{\sum }}{\left(y_{i}-\bar{y}\right)}^{2}}$$

(15) $$MSE=\frac{1}{N}\overset{N}{\underset{i=1}{\sum }}{\left(y_{i}-y_{i}^{\prime }\right)}^{2}$$

(16) $$MAE=\frac{1}{N}\overset{N}{\underset{i=1}{\sum }}\left|y_{i}-y_{i}^{\prime }\right|$$

(17) $$RMSE=\sqrt{\frac{1}{N}\overset{N}{\underset{i=1}{\sum }}{\left(y_{i}-y_{i}^{\prime }\right)}^{2}}$$

Table 9 presents the accuracy indicators of different models. LSTM has the best performance with an R^2^ of 0.965 for the training dataset and 0.909 for the testing dataset, and a low MSE of 0.04 and 0.08 for the training and testing dataset respectively. Decision Tree and Random Forest have similar performance, with a R^2^ of 0.8 for the training data set and 0.55 for the test dataset. MLP and SVM have similar performance for the training dataset, but MLP performs better for the test dataset. Decision Tree, Random Forest, and SVM models show overfitting issue with better performance for the training dataset than for the test dataset. MLP and LSTM do not show overfitting.

**Table 9.** Accuracy indicators of different models.

	Training dataset				Testing dataset			
	R^2^	MSE	MAE	RMSE	R^2^	MSE	MAE	RMSE
Decision tree	0.8695	0.1304	0.200	0.3611	0.5585	0.4374	0.4489	0.6613
RF	0.8439	0.1559	0.2459	0.3949	0.5525	0.4425	0.4604	0.6652
SVM	0.6647	0.3351	0.3298	0.5789	0.4838	0.5104	0.4606	0.7144
MLP	0.6037	0.4017	0.4527	0.6338	0.6011	0.3755	0.5013	0.6128
LSTM	0.9648	0.0403	0.1357	0.2008	0.9085	0.0799	0.2043	0.2828

Overfitting is common in machine learning and is not necessarily detrimental. The primary goal of any model is to achieve the lowest possible error on the testing dataset, ensuring strong predictive performance. In general, as model complexity increases, training error decreases. However, for the testing dataset, an optimal level of model complexity exists—beyond this point, additional complexity may reduce generalizability. The complexity of a model is often controlled by hyperparameters, as listed in Tables 4–8 for the selected models. These hyperparameters are fine-tuned using cross-validation to identify their optimal values, thereby minimizing overfitting and improving model performance.

This optimization process can be illustrated using the Random Forest model. As shown in Table 5, the optimal values for Max Depth, Min Samples Leaf, and Min Samples Split are 10, 2, and 2, respectively. To validate these choices, we compare the model’s performance across training and testing datasets while varying each of these three hyperparameters (Tables 10–12). The results confirm that the selected values yield the best predictive performance. This demonstrates that overfitting, when managed properly through hyperparameter tuning, does not necessarily degrade model performance. Instead, selecting optimal hyperparameters through cross-validation ensures that the model generalizes well while maintaining high accuracy.

**Table 10.** Performance of Random Forest when changing Max depth values.

Max depth	Training dataset				Testing dataset			
	R^2^	MSE	MAE	RMSE	R^2^	MSE	MAE	RMSE
**10(initial)**	**0.8439**	**0.1559**	**0.2459**	**0.3949**	**0.5525**	**0.4425**	**0.4604**	**0.6652**
5	0.7628	0.2371	0.2459	0.4868	0.4901	0.5041	0.5201	0.7100
20	0.8530	0.1468	0.2362	0.3832	0.5358	0.4589	0.4801	0.6774

**Table 11.** Performance of Random Forest when changing Min samples leaf values.

Min samples leaf	Training dataset				Testing dataset			
	R^2^	MSE	MAE	RMSE	R^2^	MSE	MAE	RMSE
**2(initial)**	**0.8439**	**0.1559**	**0.2459**	**0.3949**	**0.5525**	**0.4425**	**0.4604**	**0.6652**
1	0.9114	0.0885	0.1793	0.2975	0.5347	0.4600	0.4809	0.6782
5	0.6966	0.3032	0.3611	0.5507	0.4922	0.5022	0.5282	0.7086

**Table 12.** Performance of Random Forest when changing Min samples split values.

Min samples split	Training dataset				Testing dataset			
	R^2^	MSE	MAE	RMSE	R^2^	MSE	MAE	RMSE
**2(initial)**	**0.8439**	**0.1559**	**0.2459**	**0.3949**	**0.5525**	**0.4425**	**0.4604**	**0.6652**
3	0.8525	0.1474	0.2378	0.3839	0.5347	0.4600	0.4808	0.6782
5	0.8411	0.1588	0.2477	0.3985	0.5164	0.4782	0.4856	0.6915

## Importance analysis of influencing parameters on the residue tensile strength of GFRP rebar

### Definition of SHAP value

Understanding the importance of each influencing parameter on the residue tensile strength of GFRP rebar is crucial as it can guide experimental research and design, saving the cost of trial and error. In this context, Lundberg and Lee (2017), based on the principles of game theory, proposed the SHAP (Shapley additive explanation) method, compatible with machine learning models. SHAP method provides a way to explain the impact of each feature (influencing parameters in this paper) on the model’s predictions (residual tensile strength), enhancing the interpretability of balck-box models like LSTM. The SHAP value for each feature quantifies its contribution to the prediction of residual tensile strength.

The core of the SHAP method is to calculate a corresponding SHAP value for each feature variable. It uses an additive feature attribution approach to explain each SHAP value. The model’s prediction is then interpreted as the sum of the attribution values of each input feature. The specific formula is:

(18) $$f\left(x\right)=g\left(x^{\prime }\right)=\emptyset_{0}+\overset{M}{\underset{i=1}{\sum }}\emptyset_{i}x_{i}^{\prime }$$

Here, *x* is the model’s original input, and *x'* is the simplified vector of input feature. g is the explanatory model, where $g\left(x^{\prime }\right)$ represents the sum of the SHAP values of each feature and the constant. According to the accuracy and consistency requirements, $g\left(x^{\prime }\right)$ is equal to the model’s output *f(x)*. $\emptyset_{0}$ is a constant representing the model output when all inputs are missing. $\emptyset_{i}$ is the SHAP value for the corresponding feature, and M is the number of input features.

**Figure 7.**
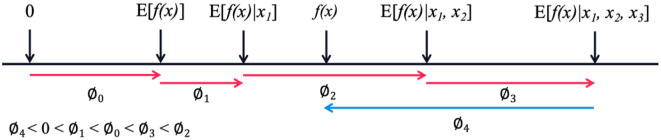
Example of calculating SHAP values.

To better understand the SHAP interpretability, Figure 7 illustrates the calculation process of the SHAP values for a sample *x = {x*_*1*_*,x*_*2*_*,x*_*3*_*,x*_*4*_*}* consisting of four features, where the prediction model result *f(x)* is computed. The process begins with the expected value of the model’s prediction *E(f(x))* as the initial constant $\emptyset_{0}$ , followed by the calculation of the SHAP values for the individual features, which are the accumulated to reach the final predicted value *f(x)*. For the sample *x*, the sum of the SHAP values equals *f(x)*. However, for each feature, the SHAP value can be either positive or negative. Blue arrow represents negative SHAP value, indicating that the corresponding feature has a negative impact on the model’s prediction; red arrows represent positive SHAP values, indicating that the corresponding features have positive impact on the model’s results. In Figure 7, features *x*_*1*_*, x*_*2*_ and *x*_*3*_ have positive effects on *f(x)*, with contributing the greatest positive impact, while feature *x*_*4*_ has a negative effect on *f(x)*.

Finally, in addition to considering the features themselves, the SHAP method takes into account all possible combinations of feature variables. Therefore, for feature $x_{i}$ , the SHAP value $\emptyset_{i}$ is equal to the weighted sum over all possible combinations of feature variables. The Equation is as follows:

(19) $$\emptyset_{i}=\underset{S\subseteq \left\{x_{1},\ldots \ldots x_{M}\right\}/\left\{x_{i}\right\}}{\sum }\frac{\left|S\right|!\left(M-\left|S\right|-1\right)!}{M!}\left[f_{x}\left(S⋃\left\{x_{i}\right\}\right)-f_{x}\left(S\right)\right]$$

Here, S is a subset of the sample feature variables, representing the set of all input features excluding $\left\{x_{i}\right\}$ . |S| denotes the number of feature variables in the subset. $f_{x}\left(S\right)$ is the model’s prediction result for the subset *S*. For more detailed information on the SHAP model, refer to Lundberg and Lee (2017).

It should be noted that, SHAP is only applicable when the influencing parameters are not highly linearly correlated. Otherwise, SHAP value cannot be used to evaluate the importance of influencing parameters. The linear correlation between influencing parameters is evaluated using Pearson Correlation Coefficient (PCC) as given in the following equation. The closer the absolute PCC value to 1, the stronger the linear correlation between two influencing parameters. A positive value indicates a positive correlation, where a negative value indicates a negative correlation.

(20) $$PCC=\frac{\overset{n}{\underset{i=1}{\sum }}\left(y_{i}-\bar{y}\right)\left(y_{i}^{\prime }-\bar{y^{\prime }}\right)}{\sqrt{\overset{n}{\underset{i=1}{\sum }}{\left(y_{i}-\bar{y}\right)}^{2}}\overset{n}{\underset{i=1}{\sum }}{\left(y_{i}^{\prime }-\bar{y^{\prime }}\right)}^{2}}$$

**Figure 8.**
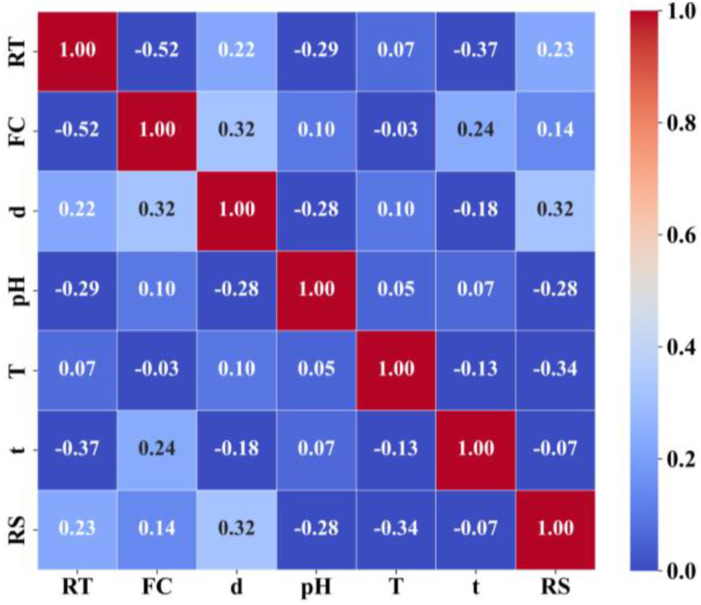
PCC results. Please note that the closer the PCC value is to 1, the stronger correlation is between two parameters. The color of the cell is a measure of PCC value as per the color scale.

PCC results are presented in Figure 8. Apart from the correlation between resin type (RT) and fiber content (FC), PCC values between other parameters are below 0.4, indicating no apparent linear correlation among these variables, ensuring the applicability of SHAP method.

PCC values can be used to assess the correlation between various influencing parameters and residual tensile strength. Exposure temperature, bar diameter, pH value, and resin type show a stronger correlation with residual tensile strength, while exposure time and fiber content have a weaker correlation. Additionally, exposure temperature and pH exhibit a negative correlation with the residual tensile strength, meaning that as temperature increases and the environment becomes more alkaline, the residual tensile strength decreases. In contrast, the bar diameter shows a positive correlation, indicating that larger diameters correspond to higher residual tensile strength, which is consistent with the underlying principles of tensile strength calculation.

The overall Pearson Correlation Coefficients among the influencing factors indicate weak inter-variable correlations, with the exception of a relatively high correlation between resin type and fiber content, showing a PCC value of −0.52. This is reasonable because resin type normally matches with particular fiber content in bar manufacturing practices.

### Importance analysis using SHAP and Random Forest

Feature selection and importance ranking methods are widely used in data processing. In engineering materials, feature importance ranking helps to identify key influencing factors, optimize material properties, and guide design and construction. Common feature selection methods include SHAP-based feature selection and model-based feature importance analysis. Both methods have been proven to effectively and accurately identify key features (Kim and Kim, 2022; Liu et al., 2022; Wang et al., 2024). The SHAP interpretability method, as a post-model approach, provides post-model explanation and analysis, independent of the prediction model type. By considering all possible combinations of feature variables, SHAP calculates the SHAP value for each feature based on the additivity principle, enabling feature importance ranking and analysis of feature interactions. In contrast, model-based feature analysis methods, such as Random Forest, analyze features during the model training process, evaluating feature importance based on the accuracy of data splits. This method focuses on the individual effect of each feature on model prediction but ignores the relationships between features. Compared to SHAP’s post-hoc analysis, model-based methods like Random Forest require fewer computational resources but are limited in scope, particularly when dealing with large feature sets or complex relationships. Research indicates that when the number of features is small (M < 15), both methods yield similar performance in terms of the area under the Precision-Recall curve (AUPRC), demonstrating their consistent effectiveness (Wang et al., 2024). However, when the number of features is large (M > 150), SHAP is more effective for identifying key features (Liu et al., 2022). In the study of GFRP rebar tensile strength, given that the feature count (M = 9) is small, both methods were used to analyze the influence of features on the residual tensile strength of GFRP rebars.

**Figure 9.**
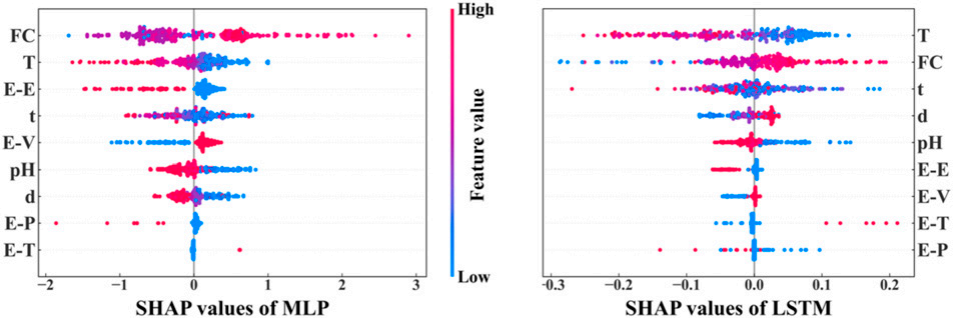
Importance ranking of influencing parameters by SHAP. Please note the spread width of the SHAP value indicates the level of importance, that is the wider the more important. The red points correspond to positive SHAP values, it means the parameter has positive impact on the residual strength, and vice versa.

Figure 9 shows the feature density scatter plot for MLP and LSTM models based on SHAP values. The influencing parameters are arranged on both sides of the figure in the order of their importance level on the residual strength of the GFRP rebar. For each influencing parameter, the spread width of the SHAP value indicates the level of importance, that is the wider the more important. If the red points correspond to positive SHAP values, it means the parameter has positive impact on the residual strength, and vice versa.

According to the results of both MLP and LSTM models, the most important parameters affecting the tensile strength of GFRP rebar are exposure temperature and fiber content. The least important parameter is the resin type. Both models show that epoxy and vinyl ester have more impact than polyester and thermoplastic. In particular, temperature has a negative impact, with residual tensile strength decreases as temperature rises. Similarly, exposure time and pH also have negative impact. Fiber content mainly shows a positive impact. These results are consistent with the experimental observations in the literature which confirms the accuracy of SHAP assessment.

**Figure 10.**
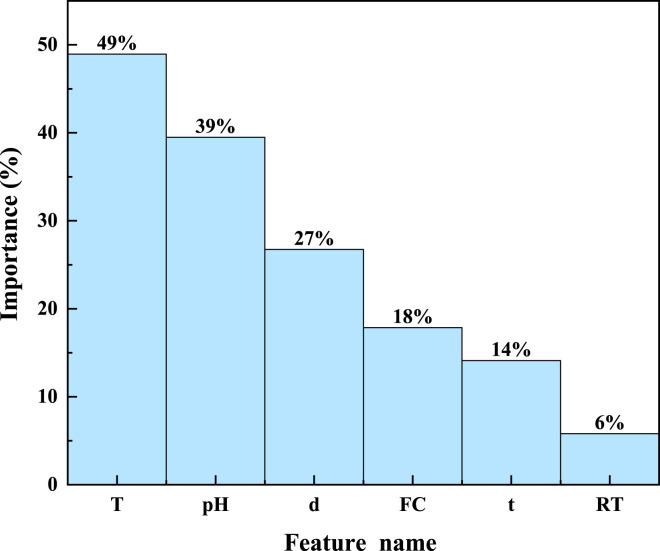
Importance ranking of influencing parameters by Random Forest.

The importance of influencing parameters is ranked using Random Forest as in Figure 10. It shows that environmental parameters (exposure temperature, pH) are generally more important than the material property parameters (bar diameter, fiber content, resin type). Similar to the SHAP results, temperature has the most significant impact on the tensile strength of GFRP rebar, followed by pH, bar diameter, fiber content, with resin type the least important. The exposure temperature has the most significant impact as confirmed by both SHAP and Random Forest which is consistent with experimental observations. For example, Feng et al. (2022) found that increased temperature lead to a significant decrease in bar tensile strength, and Al-Salloum et al. (2013) confirmed this phenomenon, discovering that GFRP rebars exposed to high temperature conditions for 18 months had an average tensile strength loss of 24.05%, while rebars in normal temperature had almost no strength loss.

Random Forest and SHAP have different mechanism which yield different importance rankings of influencing parameters. For example, Random Forest ranks pH value as the second most important parameter, but SHAP ranks pH value lower than fiber content. This indicates that pH has more noticeable impact if the other parameters do not present (Random Forest individual importance). If pH works with other parameters, its importance decreases (SHAP correlation effect). For example, it was observed that (Almusallam et al., 2013) GFRP rebars in alkaline solution with chloride ions under high temperature even showed 11% higher residual tensile strength than the bars immersed in tap water. It suggests that the coupling of pH and chloride ions reduces the impact of pH on the residual strength of GFRP rebar. This was explained (Almusallam et al., 2013; Feng et al., 2022) that a thin salt layer formed on the GFRP rebar surface, hindering water penetration leading to less strength reduction than in tap water.

In summary, temperature has the most impact on the tensile strength of GFRP rebar, both when acting alone (Random Forest) and in combination with other factors (SHAP). pH has a more noticeable impact when acting alone (Random Forest) compared to when combined with other factors (SHAP). Regarding the resin type, epoxy has a clear impact on tensile strength, while the other resin types have a smaller impact.

### Correlation analysis

**Figure 11.**
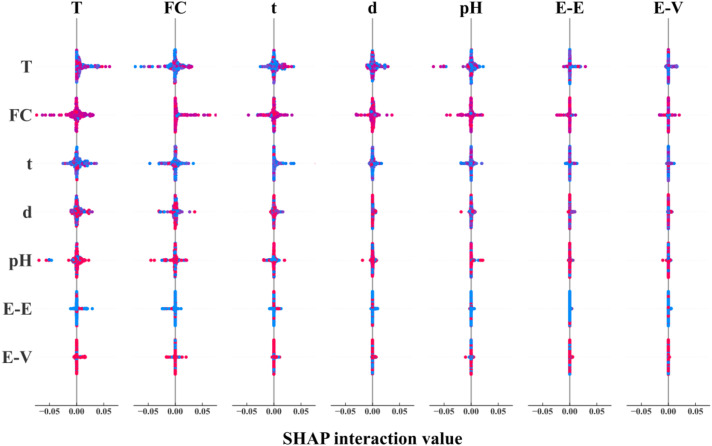
Impact of correlation between two parameters on the tensile strength of GFRP rebar using SHAP value based on LSTM model.

To further evaluate the correlation of different influencing parameters and its impact on the tensile strength of GFRP rebar, a correlation analysis is conducted based on 264 randomly selected data points (75% of database) using SHAP based on LSTM model, as shown in Figure 11. The diagonal line indicates the impact of respective parameter on the tensile strength, while off-diagonal elements represent the effect of correlation between two parameters, that is the wider the spread on the horizontal axis, the more impact the correlation generates. Figure 10 reveals that the combined effect of exposure temperature and fiber content with other parameters is the most prominent, followed by time and pH. The combined effect of resin type with other parameters is not significant.

**Figure 12.**
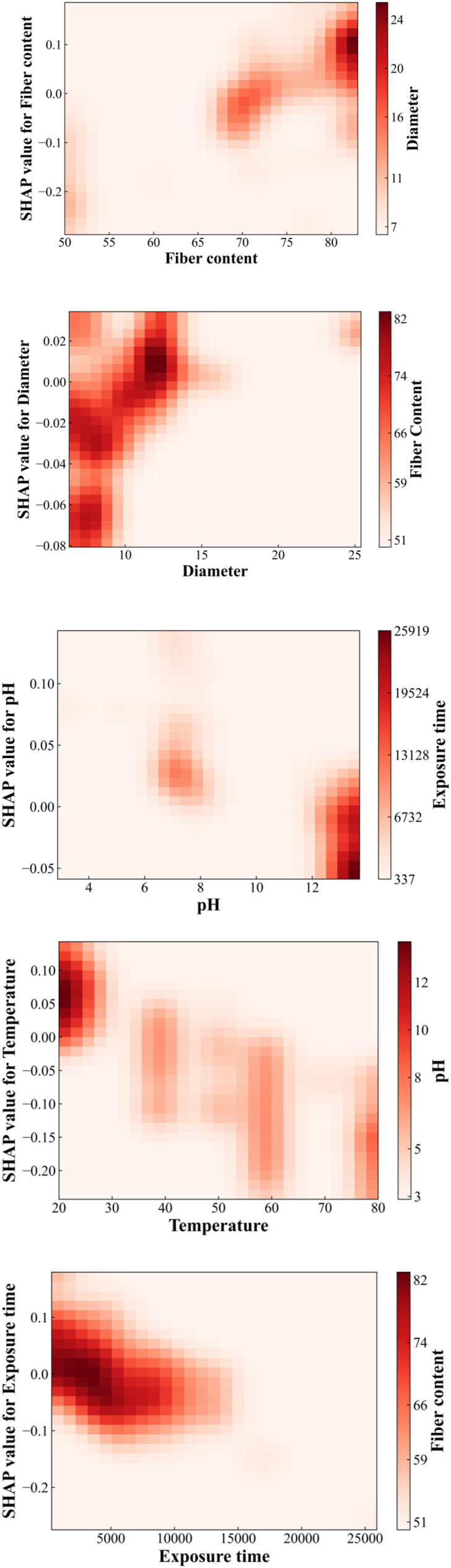
Shap correlation analysis.

Figure 12 shows the impact of individual parameters on the tensile strength within the study range and the other parameters with the most significant correlation. Where, the horizontal and vertical coordinates represent the studied parameter with its corresponding SHAP value, and the variation of SHAP value represents the trend of the parameter’s influence on the residual tensile strength, for example, in the fiber content plot, with the growth of the fiber fraction, the range of the color is gradually extended from the lower left corner (the position of SHAP values = −0.2), to the upper right corner (the position of SHAP values = 0.1), indicating that the residual tensile strength of GFRP rebar rises with increasing mass fraction. Similarly, with the increase of bar diameter, the residual tensile strength of GFRP rebar increase. In contrast, as the temperature rises and time lengthens, the residual tensile strength decreases. The residual tensile strength tends to decrease with pH value, that is when the environment is highly alkaline (pH > 12), the residual tensile strength significantly decreases, reaching its lowest at a pH of 13.7.

The right side of Figure 12 shows the parameters most correlated with the considered parameter, the depth of color represents the magnitude of correlated parameter, with darker colors indicating larger values. For example, in the fiber content plot, the deepest part of the color corresponds (d > 12 mm) to within the 80% fiber mass fraction region, and the bar diameter continues to increase as the fiber mass fraction increases, indicating higher fiber content correlates to larger bar diameter, aligning with actual engineering practices. Meanwhile, in the 75-80% fiber content range, some combinations of high fiber content with small diameter occur, predominantly with epoxy resin, suggesting that epoxy requires a lower fiber content compared to other resins. The coupling of temperature with pH occurs across the full range, and the most significant effect on the reduction of residual tensile strength of GFRP rebar is observed at low temperatures (20°C) in highly alkaline environments (pH > 12), which corresponds to the hindrance of water molecule diffusion by salt-ion films at high temperatures, as mentioned in Feng et al. (2022) and Almusallam et al. (2013) and proves that the pH effect is mainly independent. However, the pH parameter still accelerates the loss with other parameters, for example, high alkalinity can have a serious effect on the strength reduction under a long period of time.

Overall, the interdependence among the parameters is intricate. Through correlation analysis of the parameters in Figure 10, it is discovered that the mass fraction of fiber content (as a material property), and temperature (as an environmental parameter), exhibit the most significant coupling effect with other parameters. Conversely, the coupling effect between fiber type and other parameters is minimal. In the correlation analysis of continuous variables in Figure 11, it is observed that the bar diameter demonstrates the strongest positive correlation with the fiber weight fraction. Moreover, when the exposure time is short, the residual tensile strength of GFRP rebar is higher under high temperature than ambient temperature, under equivalent high alkalinity condition. Nonetheless, if the exposure time is longer than 13,000 hours, high temperature and alkaline environment introduces more tensile strength reduction of GFRP rebars.

Please note that, to ensure the reliability and robustness of the analysis of influencing parameters on the residual tensile strength of GFRP rebars, three validation approaches were employed in this section. First, domain knowledge from existing literature was used to assess the plausibility of the importance rankings, providing expert-based justification. Second, both SHAP value analysis and Random Forest-based model interpretation were applied to enable cross-validation between two different methodological perspectives in Section 5.2. The consistency/differences observed between these two approaches enhances confidence in the findings. Third, the identified key parameters were further supported by experimental evidence reported in the literature, demonstrating alignment between data-driven insights and observed physical behavior. Together, these complementary validation strategies provide a solid foundation for the interpretation of results and underscore the relevance of the identified influencing factors. Future experimental studies are encouraged to further corroborate and refine these findings.

## Conclusion

This paper presents a study to predict residual tensile strength of GFRP rebar due to environmental degradation using machine learning models, including Decision Trees, Random Forest, Support Vector Machine (SVM), Multilayer Perceptron (MLP), and Long and Short-Term Memory (LSTM). A dataset containing 350 data points on GFRP rebar tensile tests were collected from published literature, including six influencing parameters that is fiber content, bar diameter, resin type, temperature, pH, and exposure time. The importance of these parameters was also analyzed and ranked using Random Forest and SHAP in terms of their impact on the tensile strength. Based on the results in the paper, the following conclusions can be drawn:

(1) The LSTM model achieved the best performance in predicting the residual tensile strength of GFRP rebar under environmental degradation, with R^2^ values of 0.96 on the training dataset and 0.91 on the testing dataset. This was followed by the Decision Tree, Random Forest, MLP, and SVM models. However, the Decision Tree, Random Forest, and SVM models exhibited overfitting, showing better performance on the training dataset than on the testing dataset. In particular, the Decision Tree and Random Forest models had R^2^ values above 0.8 on the training set, which dropped to approximately 0.55 on the testing set.
(2) SHAP analysis indicates that temperature and fiber content are the top two most important parameters affecting GFRP rebar tensile strength, with temperature has negative and fiber content has positive effects. Additionally, fiber content is most likely coupled with other parameters and this coupling also affects the tensile strength. These results are consistent with experimental observations.
(3) Random Forest and SHAP analysis show that temperature has the most impact on the tensile strength of GFRP rebar, both when acting alone (Random Forest) and in combination with other parameters (SHAP). pH has a more noticeable impact when acting alone (Random Forest) compared to when combined with other parameters (SHAP).
(4) It is recommended that LSTM model be used for the prediction of the tensile strength of GFRP rebars due to environmental degradation. Additionally, fiber content is a very important parameter for GFRP rebar durability and it should be considered and reported in any future durability investigations.

Limited by the experimental conditions, data collection time, and available resources, this study is based on only 350 open-source tensile test data for GFRP rebars, and lacks long-term data on the variation in tensile strength of GFRP rebars in engineering applications. Additionally, due to engineering cost and demand, the fiber content, bar diameter and matrix type of GFRP rebars are generally fixed. This limits the ability to explore the coupling relationships between different feature combinations and their impact on the long-term mechanical properties. Nevertheless, the proposed predictive model and feature analysis methods still provide theoretical support for improving the tensile mechanical performance of GFRP rebars. Further research should focus on collecting more diverse long-term mechanical performance data of GFRP rebars in engineering applications to further refine related models and theories.

## Supplemental Material

Supplemental Material - Machine learning-based prediction of tensile strength of glass fiber-reinforced polymer rebar under environmental conditions Supplemental Material for Machine learning-based prediction of tensile strength of glass fiber-reinforced polymer rebar under environmental conditions by Yuqing Cheng, Xiangdong Geng, Chao Wu in Advances in Structural Engineering.

## Data Availability

The raw/processed data required to reproduce these findings cannot be shared at this time as the data also forms part of an ongoing study. *
