# Supplementary material for: Machine learning-based prediction of tensile strength of glass fiber-reinforced polymer rebar under environmental conditions
Source: Adv Struct Eng. 2025 Jul 29;29(5):831–48. doi: 10.1177/13694332251363357 (PMC13086228; doi:10.1177/13694332251363357)
Supplement: Supplemental Material - Machine learning-based prediction of tensile strength of glass fiber-reinforced polymer rebar under environmental conditions [file sj-pdf-1-ase-10.1177_13694332251363357.pdf]

Appendix

<!--Col Count:10-->fiber content (wt.%)	Diameter (mm)	pH	Temperature (°C)	Exposure time(h)	Resin type polyester	Resin type vinyl ester	Resin type epoxy	Resin type thermplastic	Residual tensile strength
70	9.53	7	60	1680	0	1	0	0	0.71
70	9.53	7	40	1680	0	1	0	0	0.97
70	9.53	7	20	2880	0	1	0	0	0.95
70	9.53	13.6	60	1680	0	1	0	0	0.64
70	9.53	13.6	40	1680	0	1	0	0	0.89
70	9.53	13.6	20	2880	0	1	0	0	0.86
70	9.53	12.7	60	1680	0	1	0	0	0.73
70	9.53	12.7	40	1680	0	1	0	0	0.92
70	9.53	12.7	20	2880	0	1	0	0	0.92
70	9.53	8	60	1680	0	1	0	0	0.74
70	9.53	8	40	1680	0	1	0	0	0.98
70	9.53	8	20	2880	0	1	0	0	0.97
70	9.53	13.6	60	1440	0	1	0	0	0.52
70	9.53	13.6	40	1440	0	1	0	0	0.69

70	9.53	13.6	20	2160	0	1	0	0	0.64
70	9.53	13.6	20	2880	0	1	0	0	0.55
70	9.53	7	20	2160	0	1	0	0	0.9
70	9.53	7	60	2160	0	1	0	0	0.61
70	9.53	13.6	20	1440	0	1	0	0	0.52
70	9.53	13.6	20	2160	0	1	0	0	0.44
70	9.53	13.6	20	2880	0	1	0	0	0.38
70	9.53	13.6	20	5760	0	1	0	0	0.23
70	9.53	13.6	40	1440	0	1	0	0	0.69
70	9.53	13.6	40	2160	0	1	0	0	0.6
70	9.53	13.6	40	2880	0	1	0	0	0.5
70	9.53	13.6	40	5760	0	1	0	0	0.33
70	9.53	13.6	60	1440	0	1	0	0	0.82
70	9.53	13.6	60	2160	0	1	0	0	0.64
70	9.53	13.6	60	2880	0	1	0	0	0.55
70	9.53	13.6	60	5760	0	1	0	0	0.43
70	9.53	12.7	20	1440	0	1	0	0	0.76
70	9.53	12.7	20	1680	0	1	0	0	0.73

70	9.53	12.7	20	2160	0	1	0	0	0.66
70	9.53	12.7	20	2880	0	1	0	0	0.59
70	9.53	12.7	40	1440	0	1	0	0	0.93
70	9.53	12.7	40	1680	0	1	0	0	0.92
70	9.53	12.7	40	2160	0	1	0	0	0.86
70	9.53	12.7	40	2880	0	1	0	0	0.86
70	9.53	12.7	60	1440	0	1	0	0	0.98
70	9.53	12.7	60	1680	0	1	0	0	0.97
70	9.53	12.7	60	2160	0	1	0	0	0.95
70	9.53	12.7	60	2880	0	1	0	0	0.92
73.3	12.7	7	25	720	0	1	0	0	0.92
73.3	12.7	7	25	1440	0	1	0	0	0.86
73.3	12.7	7	25	2160	0	1	0	0	0.8
73.3	12.7	7	25	3168	0	1	0	0	0.89
73.3	12.7	7	40	720	0	1	0	0	0.89
73.3	12.7	7	40	1440	0	1	0	0	0.85
73.3	12.7	7	40	2160	0	1	0	0	0.83
73.3	12.7	7	40	3168	0	1	0	0	0.84

73.3	12.7	7	80	720	0	1	0	0	0.85
73.3	12.7	7	80	1440	0	1	0	0	0.76
73.3	12.7	7	80	2160	0	1	0	0	0.8
73.3	12.7	7	80	3168	0	1	0	0	0.78
69.1	12.7	7	25	720	0	1	0	0	0.95
69.1	12.7	7	25	1440	0	1	0	0	0.76
69.1	12.7	7	25	2160	0	1	0	0	0.82
69.1	12.7	7	25	3168	0	1	0	0	0.88
69.1	12.7	7	40	720	0	1	0	0	0.92
69.1	12.7	7	40	1440	0	1	0	0	0.87
69.1	12.7	7	40	2160	0	1	0	0	0.78
69.1	12.7	7	40	3168	0	1	0	0	0.81
69.1	12.7	7	80	720	0	1	0	0	0.66
69.1	12.7	7	80	1440	0	1	0	0	0.55
69.1	12.7	7	80	2160	0	1	0	0	0.49
69.1	12.7	7	80	3168	0	1	0	0	0.44
73.3	12.7	8	25	720	0	1	0	0	0.93
73.3	12.7	8	25	1440	0	1	0	0	0.82

73.3	12.7	8	25	2160	0	1	0	0	0.83
73.3	12.7	8	25	3168	0	1	0	0	0.86
73.3	12.7	8	40	720	0	1	0	0	0.91
73.3	12.7	8	40	1440	0	1	0	0	0.84
73.3	12.7	8	40	2160	0	1	0	0	0.84
73.3	12.7	8	40	3168	0	1	0	0	0.87
73.3	12.7	8	80	720	0	1	0	0	0.9
73.3	12.7	8	80	1440	0	1	0	0	0.81
73.3	12.7	8	80	2160	0	1	0	0	0.8
73.3	12.7	8	80	3168	0	1	0	0	0.81
69.1	12.7	8	25	720	0	1	0	0	0.95
69.1	12.7	8	25	1440	0	1	0	0	0.84
69.1	12.7	8	25	2160	0	1	0	0	0.84
69.1	12.7	8	25	3168	0	1	0	0	0.81
69.1	12.7	8	40	720	0	1	0	0	0.9
69.1	12.7	8	40	1440	0	1	0	0	0.77
69.1	12.7	8	40	2160	0	1	0	0	0.82
69.1	12.7	8	40	3168	0	1	0	0	0.83

69.1	12.7	8	80	720	0	1	0	0	0.65
69.1	12.7	8	80	1440	0	1	0	0	0.53
69.1	12.7	8	80	2160	0	1	0	0	0.53
69.1	12.7	8	80	3168	0	1	0	0	0.43
73.3	12.7	13	25	720	0	1	0	0	0.76
73.3	12.7	13	25	1440	0	1	0	0	0.68
73.3	12.7	13	25	1800	0	1	0	0	0.66
73.3	12.7	13	40	720	0	1	0	0	0.78
73.3	12.7	13	40	1440	0	1	0	0	0.7
73.3	12.7	13	40	1800	0	1	0	0	0.67
73.3	12.7	13	80	720	0	1	0	0	0.64
73.3	12.7	13	80	1440	0	1	0	0	0.6
69.1	12.7	13	25	720	0	1	0	0	0.94
69.1	12.7	13	25	1440	0	1	0	0	0.83
69.1	12.7	13	40	720	0	1	0	0	0.87
69.1	12.7	13	40	1440	0	1	0	0	0.82
69.1	12.7	13	40	1800	0	1	0	0	0.81
69.1	12.7	13	80	720	0	1	0	0	0.63

69.1	12.7	13	80	1440	0	1	0	0	0.5
83	12	13	60	504	0	0	0	1	1
83	12	13	60	504	0	0	0	1	1.02
83	12	13	60	504	0	0	0	1	0.98
50	6.35	13	60	504	1	0	0	0	0.61
50	6.35	13	60	504	1	0	0	0	0.76
50	6.35	13	60	504	1	0	0	0	0.72
83	12	13	60	1008	0	0	0	1	0.98
83	12	13	60	1008	0	0	0	1	1.02
83	12	13	60	1008	0	0	0	1	1.02
50	6.35	13	60	1008	1	0	0	0	0.47
50	6.35	13	60	1008	1	0	0	0	0.69
50	6.35	13	60	1008	1	0	0	0	0.62
83	12	7	23	4380	0	1	0	0	1
83	12	7	23	4380	0	1	0	0	0.98
83	12	7	50	4380	0	1	0	0	0.83
83	12	8	23	4380	0	1	0	0	0.98
83	12	8	50	4380	0	1	0	0	0.89

83	12	8	50	4380	0	1	0	0	0.91
83	12	12.75	50	4380	0	1	0	0	0.85
83	12	7	50	4380	0	1	0	0	1
83	12	7	23	8760	0	1	0	0	0.99
83	12	7	23	8760	0	1	0	0	0.98
83	12	7	50	8760	0	1	0	0	0.78
83	12	8	23	8760	0	1	0	0	0.91
83	12	8	50	8760	0	1	0	0	0.91
83	12	8	50	8760	0	1	0	0	0.91
83	12	12.75	50	8760	0	1	0	0	0.8
83	12	7	50	8760	0	1	0	0	1
83	12	7	23	13140	0	1	0	0	1
83	12	7	23	13140	0	1	0	0	0.95
83	12	7	50	13140	0	1	0	0	0.76
83	12	8	23	13140	0	1	0	0	0.9
83	12	8	50	13140	0	1	0	0	0.88
83	12	8	50	13140	0	1	0	0	0.91
83	12	12.75	50	13140	0	1	0	0	0.76

83	12	7	50	13140	0	1	0	0	0.98
70	9.53	13.7	20	720	0	1	0	0	0.98
70	9.53	13.7	20	2160	0	1	0	0	0.93
70	9.53	13.7	20	3600	0	1	0	0	0.84
70	9.53	13.7	20	5040	0	1	0	0	0.82
70	9.53	13.7	20	6480	0	1	0	0	0.83
70	9.53	13.7	40	720	0	1	0	0	0.87
70	9.53	13.7	40	2160	0	1	0	0	0.8
70	9.53	13.7	40	3600	0	1	0	0	0.7
70	9.53	13.7	40	5040	0	1	0	0	0.68
70	9.53	13.7	40	6480	0	1	0	0	0.65
70	9.53	13.7	50	720	0	1	0	0	0.8
70	9.53	13.7	50	2160	0	1	0	0	0.67
70	9.53	13.7	50	3600	0	1	0	0	0.53
70	9.53	13.7	50	5040	0	1	0	0	0.51
70	9.53	13.7	50	6480	0	1	0	0	0.47
70	9.53	13.7	60	720	0	1	0	0	0.78
70	9.53	13.7	60	2160	0	1	0	0	0.61

70	9.53	13.7	60	3600	0	1	0	0	0.48
70	9.53	13.7	60	5040	0	1	0	0	0.46
70	9.53	13.7	60	6480	0	1	0	0	0.45
70	9.53	13.7	20	2160	0	1	0	0	0.89
70	9.53	13.7	20	2880	0	1	0	0	0.84
70	9.53	13.7	20	4080	0	1	0	0	0.84
70	9.53	13.7	20	5040	0	1	0	0	0.8
70	9.53	13.7	40	2160	0	1	0	0	0.84
70	9.53	13.7	40	2880	0	1	0	0	0.8
70	9.53	13.7	40	5040	0	1	0	0	0.7
70	9.53	13.7	60	2160	0	1	0	0	0.63
70	9.53	13.7	60	2880	0	1	0	0	0.6
70	9.53	13.7	60	4080	0	1	0	0	0.48
70	9.53	13.7	60	5040	0	1	0	0	0.47
70	9.53	13.7	60	2880	0	1	0	0	0.58
70	9.53	13.7	60	5040	0	1	0	0	0.48
70	9.53	13.7	23	3600	0	1	0	0	0.98
70	9.53	13.7	23	3600	0	1	0	0	0.97

70	9.53	7	23	3600	0	1	0	0	0.94
70	9.53	13.7	23	3600	0	1	0	0	0.89
70	9.53	13.7	23	3600	0	1	0	0	0.81
70	9.53	13.7	23	3600	0	1	0	0	0.95
70	9.53	7	23	3600	0	1	0	0	0.9
70	9.53	13.7	23	3600	0	1	0	0	0.89
70	9.53	13.7	23	3600	0	1	0	0	0.92
70	14	13	60	744	0	1	0	0	0.97
70	14	13	60	1488	0	1	0	0	0.99
70	14	13	60	2208	0	1	0	0	0.96
70	14	13	60	2928	0	1	0	0	0.96
70	14	13	60	4392	0	1	0	0	0.89
70	14	13	60	10248	0	1	0	0	0.87
70	14	13	60	17520	0	1	0	0	0.74
81.5	12.7	13.7	23	1440	0	1	0	0	0.96
81.5	12.7	13.7	40	1440	0	1	0	0	0.96
81.5	12.7	13.7	50	1440	0	1	0	0	0.97
81.5	12.7	13.7	23	2880	0	1	0	0	0.89

81.5	12.7	13.7	40	2880	0	1	0	0	0.85
81.5	12.7	13.7	50	2880	0	1	0	0	0.91
81.5	12.7	13.7	23	4320	0	1	0	0	0.91
81.5	12.7	13.7	40	4320	0	1	0	0	0.9
81.5	12.7	13.7	50	4320	0	1	0	0	0.9
81.5	12.7	13.7	23	5760	0	1	0	0	0.91
81.5	12.7	13.7	40	5760	0	1	0	0	0.9
81.5	12.7	13.7	50	5760	0	1	0	0	0.84
77.9	12.7	13.7	23	1440	0	1	0	0	0.99
77.9	12.7	13.7	40	1440	0	1	0	0	0.99
77.9	12.7	13.7	50	1440	0	1	0	0	0.97
77.9	12.7	13.7	23	2880	0	1	0	0	0.98
77.9	12.7	13.7	40	2880	0	1	0	0	0.96
77.9	12.7	13.7	50	2880	0	1	0	0	0.96
77.9	12.7	13.7	70	2880	0	1	0	0	0.94
77.9	12.7	13.7	23	5040	0	1	0	0	0.97
77.9	12.7	13.7	40	5040	0	1	0	0	0.95
77.9	12.7	13.7	50	5040	0	1	0	0	0.94

77.9	12.7	13.7	23	8760	0	1	0	0	0.92
77.9	12.7	13.7	40	8760	0	1	0	0	0.9
77.9	12.7	13.7	50	8760	0	1	0	0	0.89
77.9	12.7	13.7	70	2880	0	1	0	0	0.95
77.566	12.7	12.6	20	720	0	1	0	0	0.99
77.566	12.7	12.6	20	1440	0	1	0	0	0.97
77.566	12.7	12.6	20	2160	0	1	0	0	0.94
77.566	12.7	12.6	20	4320	0	1	0	0	0.87
77.566	12.7	12.6	20	5760	0	1	0	0	0.87
77.566	12.7	12.6	20	7200	0	1	0	0	0.83
77.566	12.7	12.6	40	720	0	1	0	0	0.98
77.566	12.7	12.6	40	1440	0	1	0	0	0.95
77.566	12.7	12.6	40	2160	0	1	0	0	0.92
77.566	12.7	12.6	40	4320	0	1	0	0	0.85
77.566	12.7	12.6	40	5760	0	1	0	0	0.78
77.566	12.7	12.6	40	7200	0	1	0	0	0.7
77.566	12.7	12.6	60	720	0	1	0	0	0.91
77.566	12.7	12.6	60	1440	0	1	0	0	0.86

77.566	12.7	12.6	60	2160	0	1	0	0	0.75
77.566	12.7	12.6	60	4320	0	1	0	0	0.74
77.566	12.7	12.6	60	5760	0	1	0	0	0.73
77.566	12.7	12.6	60	7200	0	1	0	0	0.69
77.566	12.7	12.6	80	720	0	1	0	0	0.82
77.566	12.7	12.6	80	1440	0	1	0	0	0.8
77.566	12.7	12.6	80	2160	0	1	0	0	0.74
77.566	12.7	12.6	80	4320	0	1	0	0	0.7
77.566	12.7	12.6	80	5760	0	1	0	0	0.7
77.566	12.7	12.6	80	7200	0	1	0	0	0.64
77.566	12.7	7	20	1200	0	1	0	0	1.05
77.566	12.7	7	20	2400	0	1	0	0	1.03
50	6.35	7	23	672	1	0	0	0	0.97
50	6.35	7	50	672	1	0	0	0	0.9
50	6.35	7	80	672	1	0	0	0	0.24
50	6.35	7	23	672	1	0	0	0	0.97
50	6.35	12	23	672	1	0	0	0	0.83
50	6.35	4.93	23	672	1	0	0	0	0.93

60	6.35	7	80	336	0	1	0	0	1.11
60	6.35	2.88	80	336	0	1	0	0	1.09
78	7.2	13.7	40	2736	0	0	1	0	0.25
78	7.2	13.7	40	2736	0	0	1	0	0.25
78	7.2	13.7	60	1800	0	0	1	0	0.25
78	7.2	13.7	60	4824	0	0	1	0	0.25
78	7.2	13.7	60	5688	0	0	1	0	0.25
78	7.2	13.7	60	5832	0	0	1	0	0.25
78	7.2	13.7	60	6912	0	0	1	0	0.25
78	7.2	13.7	60	6912	0	0	1	0	0.25
75	8	13.7	40	6192	0	0	1	0	0.25
75	8	13.7	40	9792	0	0	1	0	0.25
75	8	13.7	60	3528	0	0	1	0	0.25
75	8	13.7	60	3528	0	0	1	0	0.25
75	8	13.7	60	3456	0	0	1	0	0.25
75	8	13.7	60	6192	0	0	1	0	0.25
75	8	13.7	60	8928	0	0	1	0	0.25
78	7.2	13.7	20	3600	0	0	1	0	0.9

78	7.2	13.7	40	3600	0	0	1	0	0.84
78	7.2	13.7	60	3600	0	0	1	0	0.72
78	7.2	13.7	20	7200	0	0	1	0	0.9
78	7.2	13.7	40	7200	0	0	1	0	0.72
78	7.2	13.7	20	10800	0	0	1	0	0.84
78	7.2	13.7	40	10800	0	0	1	0	0.51
78	7.2	13.7	60	10800	0	0	1	0	0.45
75	8	13.7	20	3600	0	0	1	0	0.84
75	8	13.7	40	3600	0	0	1	0	0.63
75	8	13.7	60	3600	0	0	1	0	0.58
75	8	13.7	20	7200	0	0	1	0	0.78
75	8	13.7	40	7200	0	0	1	0	0.62
75	8	13.7	60	7200	0	0	1	0	0.42
75	8	13.7	20	10800	0	0	1	0	0.7
75	8	13.7	40	10800	0	0	1	0	0.48
75	8	13.7	60	10800	0	0	1	0	0.36
78	7.2	13.7	20	3600	0	0	1	0	0.98
78	7.2	13.7	40	3600	0	0	1	0	0.92

78	7.2	13.7	60	3600	0	0	1	0	0.9
78	7.2	13.7	20	7200	0	0	1	0	0.98
78	7.2	13.7	40	7200	0	0	1	0	0.95
78	7.2	13.7	60	7200	0	0	1	0	0.94
78	7.2	13.7	20	10800	0	0	1	0	0.98
78	7.2	13.7	40	10800	0	0	1	0	0.92
78	7.2	13.7	60	10800	0	0	1	0	0.85
75	8	13.7	20	3600	0	0	1	0	0.84
75	8	13.7	40	3600	0	0	1	0	0.77
75	8	13.7	60	3600	0	0	1	0	0.73
75	8	13.7	20	7200	0	0	1	0	0.79
75	8	13.7	40	7200	0	0	1	0	0.66
75	8	13.7	60	7200	0	0	1	0	0.5
75	8	13.7	20	10800	0	0	1	0	0.73
75	8	13.7	40	10800	0	0	1	0	0.68
75	8	13.7	60	10800	0	0	1	0	0.53
51	10.2	12.4	23	4320	0	1	0	0	0.88
51	10.2	12.4	23	11520	0	1	0	0	0.9

51	10.2	12.4	23	25920	0	1	0	0	0.92
51	10.2	12.4	60	1440	0	1	0	0	0.87
51	10.2	12.4	60	4320	0	1	0	0	0.81
51	10.2	12.4	60	9360	0	1	0	0	0.74
72	10	7	23	4320	0	1	0	0	0.98
72	10	7	23	8640	0	1	0	0	0.97
72	10	7	23	12960	0	1	0	0	0.96
72	10	7	23	4320	0	1	0	0	0.94
72	10	7	23	8640	0	1	0	0	0.9
72	10	7	23	12960	0	1	0	0	0.89
72	10	7	23	4320	0	1	0	0	0.94
72	10	7	23	8640	0	1	0	0	0.91
72	10	7	23	12960	0	1	0	0	0.87
72	10	7	23	4320	0	1	0	0	0.89
72	10	7	23	8640	0	1	0	0	0.86
72	10	7	23	12960	0	1	0	0	0.84
72	10	13.5	60	4320	0	1	0	0	0.86
72	10	13.5	60	8640	0	1	0	0	0.84

72	10	13.5	60	12960	0	1	0	0	0.8
72	10	13.5	60	4320	0	1	0	0	0.84
72	10	13.5	60	8640	0	1	0	0	0.8
72	10	13.5	60	12960	0	1	0	0	0.78
72	10	13.5	60	4320	0	1	0	0	0.83
72	10	13.5	60	8640	0	1	0	0	0.78
72	10	13.5	60	12960	0	1	0	0	0.75
80.9	9.5	12.6	60	2160	0	1	0	0	0.95
81.8	12.7	12.6	60	2160	0	1	0	0	0.87
82.6	15.9	12.6	60	2160	0	1	0	0	0.92
82.7	19.1	12.6	60	2160	0	1	0	0	0.86
83	25.4	12.6	60	2160	0	1	0	0	0.88
79	7.2	13.4	20	3600	0	0	1	0	0.97
79	7.2	13.4	40	3600	0	0	1	0	0.91
79	7.2	13.4	60	3600	0	0	1	0	0.89
79	7.2	13.4	20	7200	0	0	1	0	0.98
79	7.2	13.4	40	7200	0	0	1	0	0.94
79	7.2	13.4	60	7200	0	0	1	0	0.93

79	7.2	13.4	20	10800	0	0	1	0	0.98
79	7.2	13.4	40	10800	0	0	1	0	0.91
79	7.2	13.4	60	10800	0	0	1	0	0.84
75	8	13.4	20	3600	0	0	1	0	0.81
75	8	13.4	40	3600	0	0	1	0	0.77
75	8	13.4	60	3600	0	0	1	0	0.71
75	8	13.4	20	7200	0	0	1	0	0.78
75	8	13.4	40	7200	0	0	1	0	0.66
75	8	13.4	60	7200	0	0	1	0	0.5
75	8	13.4	20	10800	0	0	1	0	0.72
75	8	13.4	40	10800	0	0	1	0	0.68
75	8	13.4	60	10800	0	0	1	0	0.53
